# Cortistatin as a pleiotropic regulator of neuroimmune and inflammatory pathways in experimental disease: a scoping review of preclinical evidence

**DOI:** 10.1007/s10787-026-02319-x

**Published:** 2026-07-01

**Authors:** Marcelo Eduardo Cardozo, João Victor Cardoso dos Santos, Lilian Lacerda Bueno, Ricardo Toshio Fujiwara, Ana Laura Grossi de Oliveira, Cristiane Alves da Silva Menezes

**Affiliations:** 1 Postgraduate Program in Infectious Diseases and Tropical Medicine, School of Medicine, Federal University of Minas Gerais, Belo Horizonte, MG 30130-100 Brazil; 2 Department of Clinical and Toxicological Analysis, Faculty of Pharmacy, Federal University of Minas Gerais, Belo Horizonte, MG 31270-901 Brazil; 3 Department of Parasitology, Institute of Biological Sciences, Federal University of Minas Gerais, Belo Horizonte, MG 31270-901 Brazil

**Keywords:** Cortistatin, Neuroimmune regulation, Immunomodulation, Inflammation, Fibrosis, Experimental disease models

## Abstract

**Background:**

Inflammatory diseases represent a major global health burden, and current therapies frequently fail to achieve sustained control of inflammation or prevent progressive tissue damage. Cortistatin (CST), an endogenous neuropeptide structurally related to somatostatin, has emerged as a multifunctional regulator of neuroimmune and immunometabolic pathways.

**Methods:**

This scoping review was conducted in accordance with PRISMA-ScR guidelines and maps and synthesizes preclinical evidence on the biological roles and therapeutic potential of CST across experimental disease models.

**Results:**

A comprehensive literature search identified 31 preclinical studies published between 2006 and 2025 that evaluated CST administration or cortistatin deficiency. Despite substantial heterogeneity in disease models, including neurodegenerative, autoimmune, cardiovascular, fibrotic, and musculoskeletal conditions, CST consistently demonstrated protective effects. Across studies, CST reduced pro-inflammatory cytokine production, attenuated tissue damage, and improved functional outcomes and survival. Mechanistically, CST modulated key inflammatory pathways such as NF-κB signaling and NLRP3 inflammasome activation, suppressed Th1 and Th17 responses, and promoted regulatory T cell expansion through interactions with somatostatin receptors, GHSR1a, and MrgX2. Emerging translational strategies, including peptide analogues, protease-activated prodrugs, and nanoparticle-based delivery systems, have improved pharmacokinetic stability and therapeutic efficacy in experimental models.

**Conclusion:**

Collectively, these findings establish cortistatin as a consistent endogenous regulator of neuroimmune responses across diverse experimental conditions, highlighting its potential as a promising therapeutic target for immunomodulation in complex inflammatory diseases.

**Supplementary Information:**

The online version contains supplementary material available at https://doi.org/10.1007/s10787-026-02319-x.

## Introduction

Inflammation is a fundamental biological process that underlies a wide range of human diseases (Chen et al. 2018). While acute inflammation is crucial for host defense and tissue repair, dysregulated or persistent inflammatory responses can lead to deleterious conditions, including neurodegeneration, cardiovascular impairment, and autoimmunity (Furman et al. 2019). The global burden of chronic inflammatory diseases has grown substantially in recent decades, and current projections suggest that these conditions will account for an increasing proportion of global morbidity and mortality (Murray 2024; GBD 2025).

Despite decades of therapeutic development, current anti-inflammatory strategies have significant drawbacks. A large proportion of patients either do not respond adequately to available treatments or experience adverse effects that limit long-term use (Taylor et al. 2022). These limitations have driven increasing interest in endogenous regulatory systems that can coordinate immune responses without disrupting physiological balance.

Neuropeptides have gained attention as key mediators linking the nervous and immune systems within integrated regulatory networks (Carniglia et al. 2017; Gonzalez-Rey et al. 2007a). Although initially characterized in the context of neurotransmission and endocrine regulation, they are now recognized as active participants in immune control (Ganea et al. 2006; Mashaghi et al. 2016). Immune cells express receptors traditionally associated with neuronal signaling, and many neuropeptides are produced locally within inflamed tissues (Dalm et al. 2003a).

Within the neuroimmune regulatory network, cortistatin (CST), has emerged as a promising therapeutic candidate (Lecea et al. 1996). Initially identified in the cerebral cortex, CST is a cyclic peptide that is structurally similar to somatostatin and shares the conserved Phe-Trp-Lys-Thr motif, which enables binding to all five somatostatin receptors (Lecea 2005; Rubinfeld 2006). Subsequent studies have revealed its widespread expression in peripheral tissues, including circulating immune cells, the cardiovascular system, and the gastrointestinal tract (Duran-Prado et al. 2013; Dalm et al. 2003b). Activated monocytes, macrophages, and lymphocytes can synthesize CST, suggesting that it participates directly in local immune regulation (Dalm et al. 2003a).

Over the past two decades, experimental research has progressively expanded the functional scope of CST. Early studies demonstrated that CST administration significantly reduced mortality and pro-inflammatory cytokine production in models of lethal endotoxemia and sepsis (Gonzalez-Rey et al. 2007a). Subsequent investigations extended these findings to chronic inflammatory conditions, showing that CST suppresses key pro-inflammatory mediators, including TNF-α, IL-1β, IL-6, IL-12, and nitric oxide, while promoting regulatory pathways characterized by increased IL-10 production and expansion of regulatory T cells (Ganea et al. 2006; Duran-Prado et al. 2013; Gonzalez-Rey et al. 2006a).

Despite the growing body of evidence, no comprehensive effort has systematically mapped and critically appraised the full spectrum of CST functions across experimental disease models. The heterogeneity across animal models, peptide isoforms, administration routes, and outcome measures limits comparability between studies and hinders translational interpretation. Given the pleiotropic role of CST across immune, neural, and metabolic pathways, it represents a unique candidate for integrative anti-inflammatory strategies. Accordingly, although structured as a scoping review, this study emphasizes mechanistic convergence and translational relevance across experimental models. Therefore, the present review aims to systematically map the available evidence, identify convergent mechanistic pathways, evaluate methodological approaches, and highlight key knowledge gaps to inform future research on CST-based immunomodulatory strategies (O'Neill and Pearce 2016).

## Methods

### Protocol and guidelines

This scoping review was conducted in accordance with the PRISMA-ScR (Preferred Reporting Items for Systematic Reviews and Meta-Analyses Extension for Scoping Reviews) guidelines. The protocol was prospectively developed and registered on the Open Science Framework (OSF) (https://osf.io/xjcdr), where the research question, eligibility criteria, information sources, and approach for evidence mapping and synthesis were predefined.

### Research question

The guiding question for this scoping review was: “What is the role of cortistatin in experimental disease models in terms of its pathophysiology and therapeutic potential?” The question was formulated using the PCC (Population, Concept, Context) framework: *Population*: animal models of disease; *Concept*: cortistatin (either endogenous expression or exogenous administration); *Context*: preclinical studies across diverse experimental disease models.

### Search strategy

A comprehensive literature search was conducted in PubMed/MEDLINE, Embase, Scopus, and Web of Science from database inception to February 2026. The search strategy combined descriptors related to CST and experimental disease models using Boolean operators: (“cortistatin” OR “CST”) AND (“inflammation” OR “autoimmune” OR “fibrosis” OR “neurodegeneration” OR “disease model” OR “animal model”). The full search strategy for each database is provided in the Additional Table 1.

**Table 1:** General characteristics of the included studies that evaluated cortistatin in experimental disease models

No	Study (Country)	Main objective	Animal model	Disease model	CST intervention
1	Castillo-González et al., (2024) (Spain)	Neuroprotective effects after ischemic stroke	Mouse (C57BL/6 J)	MCAO ischemic stroke	CST-29; i.p.; post-reperfusion
2	Duan et al., (2024) (China)	CST modulation of septic cardiomyopathy	Mouse (C57BL/6 J; WT/NLRP3^−/−^)	LPS-induced sepsis	CST; i.p
3	Serrano-Martínez et al., (2024) (Spain)	Neuroprotection in Parkinson’s disease	Mouse (C57BL/6)	MPTP Parkinson model	CST-29; i.p
4	Gao et al., (2024) (China)	CST effects in osteonecrosis	Rat (Sprague–Dawley)	Osteonecrosis of the femoral head	Recombinant CST; i.p
5	Castillo-González et al., (2023) (Spain)	CST regulation of BBB dysfunction	Mouse (C57BL/6 J; WT/Cort^−/−^)	LPS-induced BBB dysfunction	CST-29
6	Mehrabi Nasab et al., (2023) (Iran)	CST-nanoparticles in allergic asthma	Mouse (Balb/c)	OVA-induced asthma	CST-gold nanoparticles; inhalation
7	Barriga et al., (2022) (Spain)	CST role in dermal and pulmonary fibrosis	Mouse (C57BL/6; CST^−/−^ and CST^±^)	Systemic Sclerosis (Bleomycin-induced)	CST-29 (s.c.); endogenous deficiency
8	Campos-Salinas et al., (2022) (Spain)	Evaluation of latent CST prodrug	Mouse (C57BL/6; Balb/c)	Sepsis, IBD, fibrosis	Latent CST-29
9	Benitez et al., (2022) (Spain)	CST in liver fibrosis progression	Mouse (C57BL/6 J; Cort^±^)	CCl₄/BDL fibrosis	CST-29; i.p
10	Cao et al., (2022) (China)	CST in osteolysis and TNFR signaling	Mouse (C57BL/6;TNFR^−/−^)	Periprosthetic osteolysis	Recombinant CST
11	Wen et al., (2022) (China)	CST protection in septic encephalopathy	Mouse (C57BL/6)	CLP-induced SAE	CST-14; i.p
12	Barriga et al., (2021) (Spain)	CST in lung injury and fibrosis	Mouse (C57BL/6; Cort^−/−^)	ALI/Bleomycin fibrosis	CST-29
13	Falo et al., (2021) (Spain)	CST effects in neuropathic pain	Mouse (C57BL/6; CST^−/−^)	Neuropathic/diabetic pain	CST-29
14	Rol et al., (2021) (Spain)	CST analogue in inflammatory bowel disease	Mouse (Balb/c)	TNBS/DSS colitis	CST analogue
15	Zhao et al., (2020) (China)	CST in disc degeneration	Rat (Sprague–Dawley)	Intervertebral disc degeneration	CST
16	Balbaba et al., (2020) (Turkey)	CST in endotoxin-induced uveitis	Rat (Wistar)	LPS-induced uveitis	CST; i.p
17	Qiu et al., (2020) (China)	CST deficiency in allergic asthma	Mouse (C57BL/6; Cort^−/−^)	OVA-induced asthma	CST deficiency
18	Zhao et al., (2019) (China)	CST regulation of osteoarthritis	Mouse (C57BL/6; Cort^−/−^)	DMM osteoarthritis	CST; intra-articular
19	Chai et al., (2018) (China)	CST effects on aortic aneurysm	Mouse (ApoE^−/−^)	AngII-induced AAA	CST; i.p
20	Delgado-Maroto et al., (2017) (Spain)	CST modulation of atherosclerosis	Mouse (ApoE^−/−^)	Atherosclerosis	CST
21	Delgado-Maroto et al., (2017) (Spain)	CST in autoimmune myocarditis	Mouse (Balb/c)	Experimental myocarditis	CST-29
22	Morell et al., (2014) (Spain)	CST in inflammatory pain	Mouse (C57BL/6)	Formalin/CFA pain	CST-29
23	Duran-Prado et al., (2013) (Spain)	Vascular CST role in SMC function and neointima formation	Mouse (C57BL/6; CST^−/−^)	Vascular Stenosis (Carotid Artery Ligation)	CST-14 (i.p.); endogenous deficiency
24	Morell et al., (2013) (Spain)	CST in arthritic pain	Mouse (C57BL/6)	Zymosan arthritis	CST-29
25	Souza-Moreira et al., (2013) (Spain)	CST in the multiple sclerosis model	Mouse (C57BL/6; SJL/J)	EAE	CST-29
26	Zhang et al., (2015) (China)	CST protection in sepsis	Rat (Sprague–Dawley)	CLP sepsis	CST-14
27	Chiu et al., (2011) (Taiwan)	CST in bacterial meningoencephalitis	Rat (Wistar)	S. pneumoniae infection	CST-14
28	Wang et al., (2008) (China)	CST in transplant rejection	Mouse (Balb/c; C57BL/6)	Skin allograft	CST
29	González-Rey et al. 2007a, 2007b (Spain)	CST in autoimmune arthritis	Mouse (DBA/1)	Collagen-induced arthritis	CST-29
30	González-Rey et al., (2006) (Spain)	CST in sepsis models	Mouse (C57BL/6)	LPS/CLP sepsis	CST-29
31	González-Rey et al., (2006) (Spain)	CST in inflammatory bowel disease	Mouse (Balb/c)	TNBS colitis	CST-29

AAA: Abdominal aortic aneurysm; Akt: Protein kinase B; ALI: Acute lung injury; AMPK: AMP-activated protein kinase; Ang II: Angiotensin II; ApoE^−/−^: Apolipoprotein E-deficient mice; BALB/c: Inbred mouse strain; BBB: Blood–brain barrier; BDL: Bile duct ligation; CFA: Complete Freund’s adjuvant; CCI: Chronic constriction injury; CCl₄: Carbon tetrachloride; CLP: Cecal ligation and puncture; CNS: Central nervous system; CPZ: Cuprizone; CST: Cortistatin; CST-14/CST-29: 14- and 29-amino acid cortistatin peptides; DMM: Destabilization of the medial meniscus; DSS: Dextran sulfate sodium; EAE: Experimental autoimmune encephalomyelitis; EAM: Experimental autoimmune myocarditis; GC: Glucocorticoid; HD: Huntington’s disease; IBD: Inflammatory bowel disease; i.a.: Intra-articular; i.c.v.: Intracerebroventricular; i.n.: Intranasal; i.p.: Intraperitoneal; i.pl.: Intraplantar; i.t.: Intrathecal; LPS: Lipopolysaccharide; MCAO: Middle cerebral artery occlusion; MPS: Methylprednisolone; MPTP: 1-methyl-4-phenyl-1,2,3,6-tetrahydropyridine; MS: Multiple sclerosis; OA: Osteoarthritis; OGD-R: Oxygen–glucose deprivation and reoxygenation; ONFH: Osteonecrosis of the femoral head; OVA: Ovalbumin; PD: Parkinson’s disease; SAE: Sepsis-associated encephalopathy; S.C: subcutaneous; SD: Sprague–Dawley rat; SMC: smooth muscle cells; SJL/J: Inbred mouse strain; SNI: Spared nerve injury; STZ: Streptozotocin; TNBS: 2,4,6-trinitrobenzenesulfonic acid; TNFR1/TNFR2^−/−^: Tumor necrosis factor receptor 1 or 2-deficient mice; WT: Wild type.

### Eligibility criteria

Studies were included if they met the following criteria: (i) original experimental studies conducted in animal models; (ii) evaluation of exogenous CST or endogenous deficiency (Cort^⁻/⁻^ models); (iii) investigation of inflammatory, autoimmune, cardiovascular, neurodegenerative, or fibrotic disease models; and (iv) reporting outcomes related to inflammation, tissue damage, organ function, or survival. Only articles published in English were considered. Studies were excluded if they were: (i) in vitro, in silico, or exclusively cell-based studies; (ii) human studies; (iii) reviews, editorials, comments, or letters; (iv) studies without original experimental data; or (v) studies that did not directly evaluate CST.

### Study selection

Records identified from database searches were imported into the Rayyan platform (Qatar Computing Research Institute) for reference management and duplicate removal. Two reviewers (MEC and JVCS) independently screened titles and abstracts using a blinded selection process. Potentially eligible studies were then assessed in full text for final inclusion. Disagreements were resolved by consensus or by consultation with a third reviewer (ALGO).

### Data charting

Data were charted in a standardized manner by two independent reviewers using a predefined extraction form. The following variables were collected: author and year of publication; country of origin; animal species and strain; experimental disease model; type of intervention (exogenous CST or endogenous deficiency); molecular form of cortistatin (e.g., CST-14, CST-29, analogues, prodrugs); route and regimen of administration; main outcomes; and reported mechanisms of action.

### Risk of bias assessment and data synthesis

Two reviewers (MEC and JVCS) independently assessed methodological quality and risk of bias, with disagreements resolved by consensus or third-party arbitration (ALGO). For preclinical studies, risk of bias was evaluated using the Collaborative Approach to Meta-Analysis and Review of Animal Experimental Studies (CAMARADES) checklist. Studies were assessed across ten methodological domains, including peer-reviewed publication, randomization, blinded outcome assessment, sample size calculation, and compliance with animal welfare regulations.

Data management and curation were performed using Microsoft Excel (Microsoft Corp.). To support the identification of mechanistic patterns across studies, an alluvial diagram was generated in R (RStudio/Posit, version 2025.05.0). Data processing and visualization were conducted using the dplyr (v1.1.4), ggplot2 (v3.5.2), and ggalluvial (v0.12.5) packages. Given the heterogeneity of experimental models, results were synthesized descriptively with emphasis on convergent immunological and molecular mechanisms.

## Results

### Main characteristics of the included studies

**Fig. 1:**
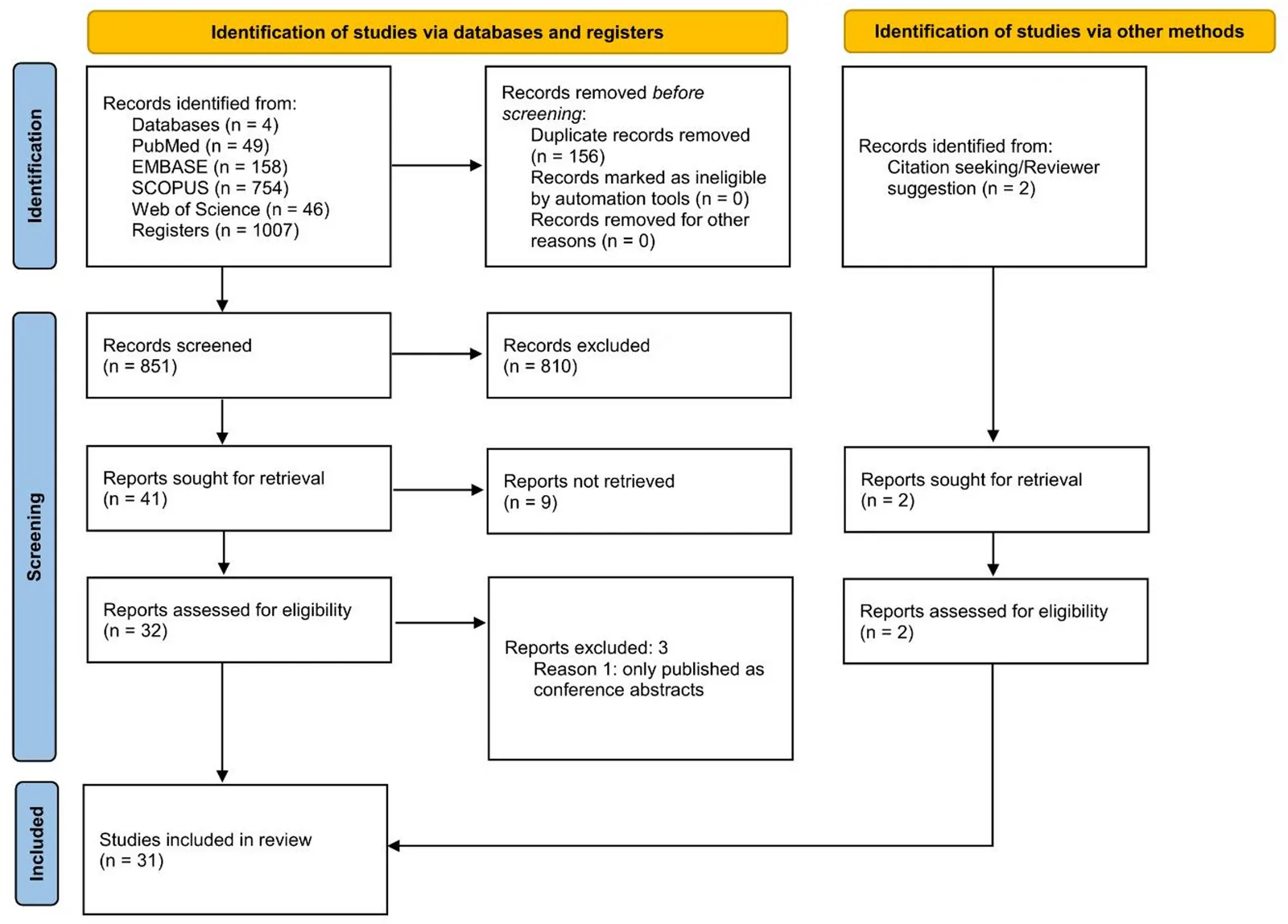
PRISMA flow chart of the study selection and inclusion process

A total of 31 preclinical studies published between 2006 and 2025 were included, with over half conducted after 2020 (n = 18; 58.1%), indicating a recent expansion of interest in CST biology. The study selection process is presented in Fig. 1 (PRISMA flow diagram), and detailed characteristics are summarized in Table 1.

Geographically, research activity was predominantly concentrated in Spain (n = 20; 64.5%) and China (n = 11; 35.4%), with limited contributions from other regions: Iran (n = 1; 3.1%), Turkey (n = 1; 3.1%), and Taiwan (n = 1; 3.1%), highlighting a geographical clustering of CST research. Most studies were interventional (n = 24; 77.4%), evaluating the therapeutic effects of exogenous CST, whereas a smaller subset employed genetic deficiency models (Cort^⁻/⁻^; n = 3; 9.7%) or combined both approaches (n = 4; 12.9%).

Across studies, murine models predominated (n = 31; 100%), primarily mice (n = 26; 83.9%), while a smaller proportion employed rat models, with diverse strains and genetically modified backgrounds enabling both mechanistic and therapeutic investigations.

### Immunomodulatory role of endogenous cortistatin and effects of CSt administration

Observational studies using Cort^⁻/⁻^ models demonstrated that CST deficiency leads to exacerbated inflammatory responses and increased tissue vulnerability, particularly in models of pulmonary fibrosis, osteoarthritis, allergic asthma, and blood–brain barrier dysfunction (Qiu et al. 2020; Barriga et al. 2021, 2022; Zhao et al. 2019; Castillo-González et al. 2023; Benitez et al. 2022).

Interventional studies consistently demonstrated that CST administration exerts robust anti-inflammatory and tissue-protective effects across diverse experimental disease models, including sepsis, fibrosis, cardiovascular disorders, neurodegeneration, musculoskeletal diseases, and autoimmune conditions (Gonzalez-Rey et al. 2006a, 2006b, 2007b; Barriga et al. 2021, 2022; Zhao et al. 2019, 2020; Castillo-González et al. 2023, 2024; Wang et al. 2008; Rol et al. 2021; Souza-Moreira et al. 2013; Zhang et al. 2015; Chai et al. 2018; Cao et al. 2022; Gao et al. 2024; Balbaba et al. 2020; Falo et al. 2021; Campos-Salinas et al. 2022; Wen et al. 2022; Benitez et al. 2022; Mehrabi Nasab et al. 2023; Serrano-Martínez et al. 2024; Duan et al. 2024; Delgado-Maroto et al. 2017a, 2017b; Morell et al. 2013, 2014; Chiu et al. 2011). These effects included reduced production of pro-inflammatory cytokines, attenuation of tissue damage, and improved functional outcomes and survival.

In systemic inflammation models, including sepsis and endotoxemia, CST reduced cytokine production, organ dysfunction, and mortality (Gonzalez-Rey et al. 2006a; Zhang et al. 2015; Campos-Salinas et al. 2022; Wen et al. 2022; Duan et al. 2024). In chronic inflammatory and autoimmune diseases, such as arthritis, colitis, and experimental autoimmune encephalomyelitis, CST suppressed Th1/Th17 responses and enhanced regulatory pathways, resulting in decreased disease severity (Gonzalez-Rey et al. 2006a, 2007b; Rol et al. 2021; Souza-Moreira et al. 2013; Delgado-Maroto et al. 2017b).

CST also demonstrated anti-fibrotic effects, reducing extracellular matrix deposition and fibroblast activation in hepatic, pulmonary, and cutaneous fibrosis (Barriga et al. 2021, 2022; Campos-Salinas et al. 2022; Benitez et al. 2022). In the central nervous system, CST exerted neuroprotective effects, including reduced glial activation, preservation of blood–brain barrier integrity, and decreased neuronal loss (Souza-Moreira et al. 2013; Wen et al. 2022; Serrano-Martínez et al. 2024; Duan et al. 2024; Morell et al. 2014). Additionally, CST exhibited analgesic properties in inflammatory and neuropathic pain models (Falo et al. 2021; Morell et al. 2013, 2014) and protective effects in musculoskeletal disorders (Cao et al. 2022; Zhao et al. 2020; Benitez et al. 2022).

### Experimental models and biological systems

The included studies employed a diverse range of experimental models and animal systems, reflecting the broad applicability of CST across inflammatory and degenerative conditions. Most studies were conducted in mice (n = 27; 87.1%), with a smaller proportion using rats (n = 5; 16.1%). Frequently used murine strains included C57BL/6, BALB/c, DBA/1, and SJL/J, alongside genetically modified models such as Cort^⁻/⁻^, ApoE^⁻/⁻^, and NLRP3^⁻/⁻^, enabling both mechanistic and functional investigation of CST signaling pathways.

This diversity of experimental systems enabled the evaluation of CST both as an endogenous regulator and as a therapeutic agent across multiple biological contexts. Studies in rats predominantly employed Sprague–Dawley and Wistar strains, particularly in models of musculoskeletal degeneration, sepsis, and ocular inflammation. Most experiments were performed in young-adult animals (6–12 weeks), although aged models were selectively used to investigate neurodegenerative and age-associated inflammatory processes.

The disease models encompassed a broad spectrum of inflammatory and degenerative conditions, with a predominance of neurological (n = 6; 19.4%) and autoimmune/inflammatory diseases (n = 8; 25.8%), including ischemic stroke, Parkinson’s disease, encephalomyelitis, arthritis, and colitis. Additional models included musculoskeletal degeneration (n = 4; 12.9%), fibrosis (n = 4; 12.9%), systemic inflammation and sepsis (n = 3; 9.6%), cardiovascular diseases (n = 3; 9.6%), transplant rejection (n = 1; 3.1%), and pain models (n = 3; 9.6%). Collectively, these models highlight the pleiotropic involvement of CST across interconnected pathophysiological pathways, including immune regulation, neuroinflammation, tissue remodeling, and systemic inflammatory responses.

**Fig. 2:**
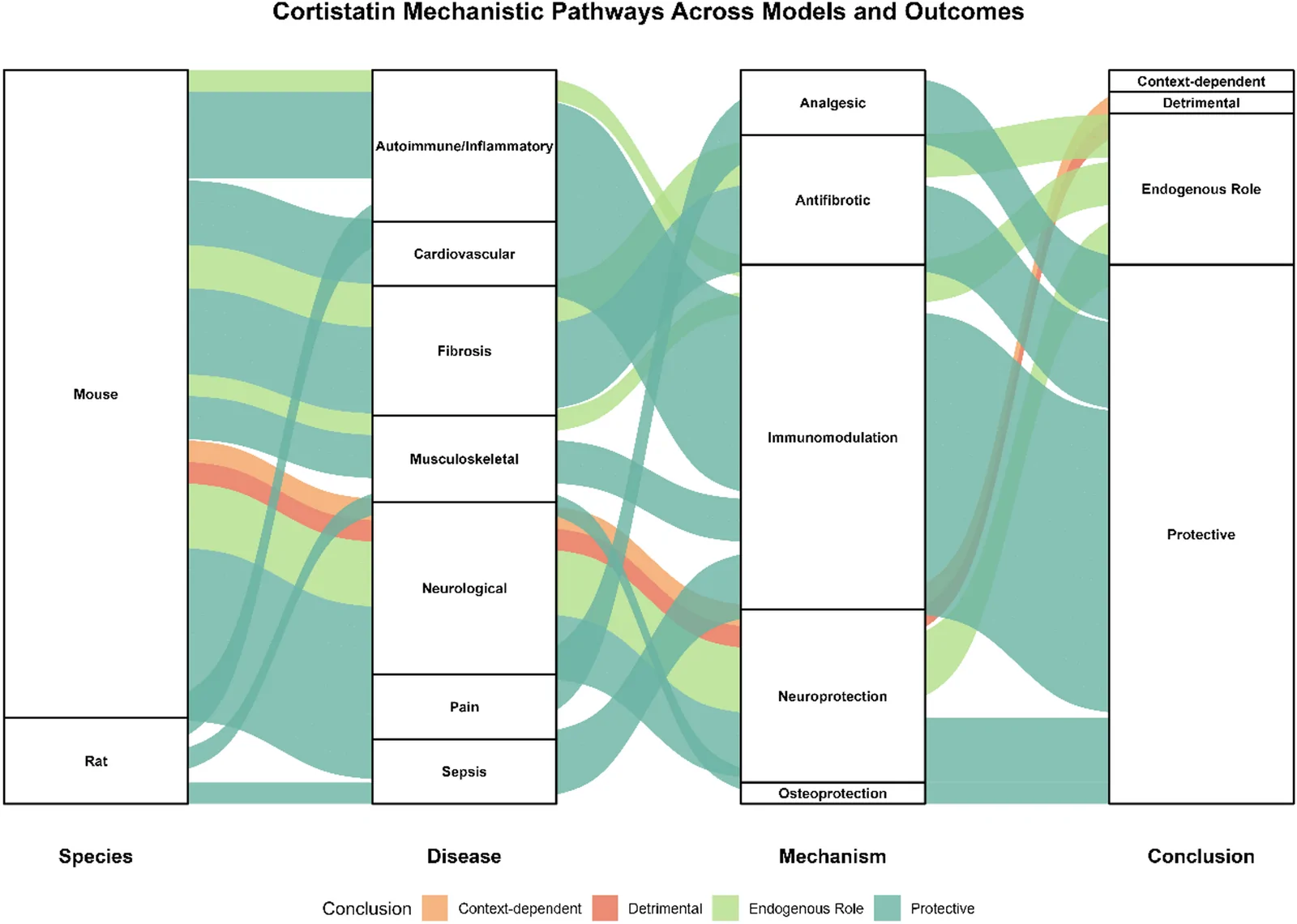
Cortistatin mechanistic pathways and functional outcomes across experimental disease models. This alluvial plot illustrates the flow of experimental evidence from (1) the animal species, (2) the disease context, (3) the primary mechanism, and (4) the study conclusions. Each stream represents the data from one or more studies reporting a specific mechanistic association. This layout offers a clear summary of how cortistatin functions across a wide range of pathological conditions. The colors represent the final interpretation of the peptide’s role, distinguishing between protective effects in teal, its essential endogenous role in light green, detrimental outcomes in red, and results that were context-dependent in orange

Across these experimental contexts, outcomes were consistently assessed at multiple biological levels. Inflammatory parameters were universally reported (n = 31; 100%), including cytokine production, cellular infiltration, and glial activation. Histopathological analyses (n = 20; 64.5%) and functional or behavioral assessments (n = 15; 48.3%) were also frequently included, while survival and pain outcomes were less commonly evaluated. This multilevel outcome framework enabled an integrated characterization of CST effects, spanning molecular signaling, tissue integrity, and functional performance (Fig. 2).

### CST formulations and therapeutic administration strategies

CST was administered in multiple molecular formats and therapeutic strategies, reflecting both mechanistic exploration and progressive efforts toward translational optimization. Native peptides, particularly CST-29 (n = 15; 48.3%), were the most frequently used, followed by CST-14 and recombinant forms. Recent studies have incorporated advanced pharmacological approaches, including synthetic analogues, nanoparticle-based delivery systems, and matrix metalloproteinase (MMP)-activated prodrugs, indicating a shift toward improved stability, targeting, and therapeutic efficacy (Campos-Salinas et al. 2022; Mehrabi Nasab et al. 2023; Castillo-González et al. 2024).

In several studies, CST isoforms were not specified, while others focused exclusively on endogenous CST function without exogenous administration (n = 2; 6.3%).

Regarding administration, the intraperitoneal route predominated (n = 26; 83.87%), consistent with its widespread use in preclinical models. Alternative routes, including intranasal, intrathecal, intra-articular, and intracerebroventricular delivery, were employed in specific experimental contexts to target distinct tissue compartments, particularly within the central nervous system.

Most studies adopted therapeutic (post-induction) regimens (n = 29; 93.5%), while a smaller proportion used prophylactic approaches. CST was administered as single or repeated doses, initiated at different stages of disease progression, reflecting heterogeneous intervention strategies and ongoing efforts to optimize pharmacological delivery across experimental settings.

### Temporal and methodological evolution of CST research

The temporal distribution of studies (2006–2025) reveals a progressive methodological and conceptual evolution in CST research. Early investigations (2006–2011) primarily focused on acute inflammatory models, typically employing CST-29 administered via intraperitoneal routes, with emphasis on cytokine modulation and survival outcomes. From 2015 onwards, studies expanded to include chronic, autoimmune, and degenerative conditions, alongside the incorporation of genetically modified models and more diverse outcome measures, including histopathological and functional endpoints.

More recent investigations (2020–2025) have increasingly adopted advanced pharmacological strategies, such as synthetic analogues, prodrugs, and nanoparticle-based delivery systems, reflecting a shift from predominantly mechanistic studies toward more translationally oriented approaches. These temporal trends provide a framework for interpreting the progressive mechanistic convergence of CST effects across experimental systems.

### Mechanistic landscape of cortistatin across experimental models

To synthesize the mechanistic evidence across studies, findings were organized into major biological domains encompassing CST immunomodulatory, neuroprotective, tissue-specific, and translational effects. The included studies consistently demonstrated a predominantly protective and pleiotropic role of CST across diverse experimental disease models, with convergent effects on inflammation control, tissue preservation, and improved functional outcomes (Gonzalez-Rey et al. 2006a; Wang et al. 2008; Rol et al. 2021; Souza-Moreira et al. 2013; Zhang et al. 2015; Chai et al. 2018; Cao et al. 2022; Zhao et al. 2020; Balbaba et al. 2020; Falo et al. 2021; Campos-Salinas et al. 2022; Wen et al. 2022; Benitez et al. 2022; Barriga et al. 2022; Mehrabi Nasab et al. 2023; Castillo-González et al. 2024; Serrano-Martínez et al. 2024; Duan et al. 2024; Delgado-Maroto et al. 2017a, 2017b; Morell et al. 2014).

Conversely, CST deficiency was associated with exacerbated inflammation, increased tissue vulnerability, and worsened disease outcomes, supporting its role as a key endogenous regulator (Barriga et al. 2021; Zhao et al. 2019; Castillo-González et al. 2023; Balbaba et al. 2020; Benitez et al. 2022). A single study reported a paradoxical phenotype associated with systemic neuroendocrine alterations rather than intrinsic immune dysregulation (Morell et al. 2013) Table 2.

### Core immunomodulatory mechanisms of CST

Across models, CST consistently modulated innate and adaptive immune responses, suppressing pro-inflammatory cytokine production and inhibiting pathogenic Th1 and Th17 responses while promoting regulatory T cell expansion. These effects were central to disease attenuation in sepsis, colitis, arthritis, and autoimmune encephalomyelitis (Gonzalez-Rey et al. 2006a; Wang et al. 2008; Campos-Salinas et al. 2022; Mehrabi Nasab et al. 2023).

**Fig. 3:**
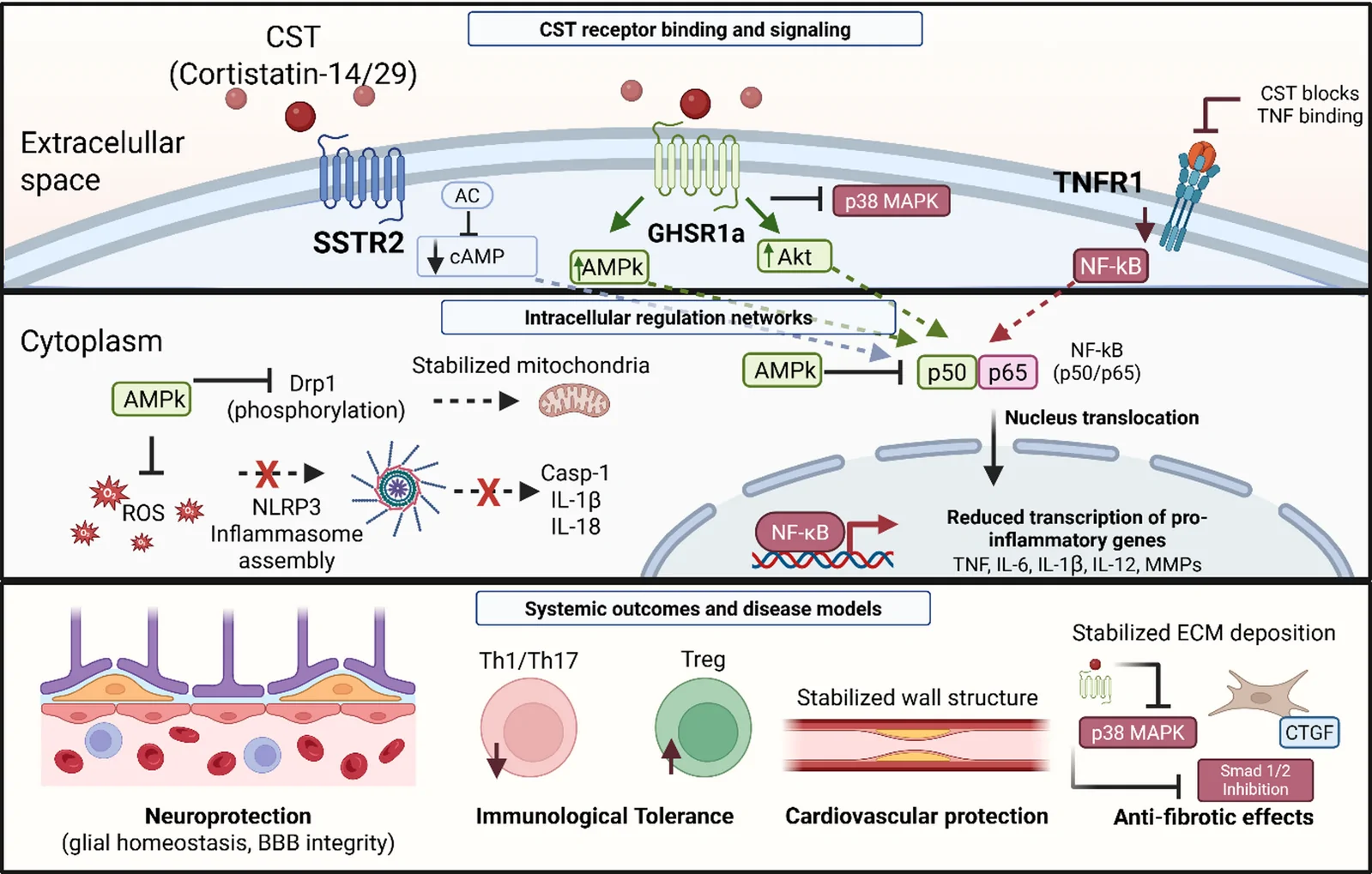
Pleiotropic signaling networks and integrated immunomodulatory and antifibrotic mechanisms of cortistatin in systemic and tissue-specific inflammation. Cortistatin (CST) exerts multifaceted protective effects through the integration of convergent signaling pathways across distinct cellular compartments. (Top panel) CST binding to SSTR2 and GHSR1a receptors modulates secondary messengers, resulting in the reduction of intracellular cAMP levels and the activation of AMPK and Akt kinases. Simultaneously, CST acts as a functional antagonist of TNFR1, impeding TNF-mediated pro-inflammatory signaling. Notably, CST-mediated GHSR1a signaling leads to the inhibition of p38 MAPK. (Middle panel) AMPK activation inhibits Drp1 phosphorylation, thereby stabilizing mitochondrial homeostasis and mitigating the production of reactive oxygen species (ROS). This prevents NLRP3 inflammasome assembly and the subsequent release of inflammatory mediators. Concurrently, CST reduces the nuclear translocation of NF-κB subunits (p50/p65), leading to reduced transcription of pro-inflammatory genes. (Bottom panel) These mechanisms converge toward favorable systemic outcomes, including neuroprotection, expansion of regulatory T cells (Tregs), and stabilization of vascular wall structures. Specifically, the inhibition of the p38 MAPK/Smad1/2 signaling axis suppresses fibroblast activation and CTGF (Connective tissue growth factor) expression, thereby mediating the observed anti-fibrotic effects

CST also inhibited key inflammatory signaling pathways, including NF-κB signaling and NLRP3 inflammasome activation, contributing to reduced systemic inflammation and organ damage (Fig. 3) (Zhang et al. 2015; Wen et al. 2022; Delgado-Maroto et al. 2017a).

### Neuroimmune and neuroprotective effects

CST exerted robust neuroprotective effects across models of stroke, Parkinson’s disease, meningitis, and sepsis-associated encephalopathy, reducing microglial activation, preserving blood–brain barrier integrity, and limiting peripheral immune cell infiltration (Souza-Moreira et al. 2013; Wen et al. 2022; Duan et al. 2024; Morell et al. 2014). Endogenous CST deficiency further revealed its role in maintaining neuroimmune homeostasis, as evidenced by increased gliosis and impaired remyelination in deficient models (Barriga et al. 2021).

### Tissue-specific protective effects: cardiovascular and musculoskeletal systems

In cardiovascular models, Duran-Prado et al. (2013) identified that both human and murine SMCs constitutively express and secrete CST, a process upregulated under pathological conditions such as arterial injury. CST reduced atherosclerotic plaque formation and vascular inflammation, including inhibition of foam cell formation through enhanced cholesterol efflux in macrophages, alongside increased regulatory T cell responses (Delgado-Maroto et al. 2017a).

In abdominal aortic aneurysm, CST reduced lesion severity through decreased macrophage infiltration, matrix metalloproteinase activity, and smooth muscle cell apoptosis, mediated by inhibition of the ERK1/2 signaling pathway (Chai et al. 2018).

In musculoskeletal diseases, CST preserved tissue integrity by reducing inflammation and apoptosis, involving TNFR1-ROS-caspase-3 and GHSR1a-Akt signaling pathways, as well as inhibition of mitochondrial NLRP3 inflammasome activation (Cao et al. 2022; Zhao et al. 2020; Benitez et al. 2022; Delgado-Maroto et al. 2017b).

### Fibrosis, tissue remodeling, and repair

CST also demonstrated anti-fibrotic effects, reducing extracellular matrix deposition and fibroblast activation in hepatic, pulmonary, and cutaneous fibrosis models (Barriga et al. 2021, 2022; Campos-Salinas et al. 2022; Benitez et al. 2022).

### Translational modulation and advanced therapeutic strategies

Advanced formulations, including matrix metalloproteinase (MMP)-activated prodrugs and controlled-release delivery systems, enhanced CST efficacy by enabling localized delivery in inflamed or fibrotic tissues (Mehrabi Nasab et al. 2023). These strategies reflect a transition toward translationally optimized CST-based therapies aimed at improving pharmacokinetic stability and tissue-specific targeting.

### Risk of bias and methodological considerations

**Fig. 4:**
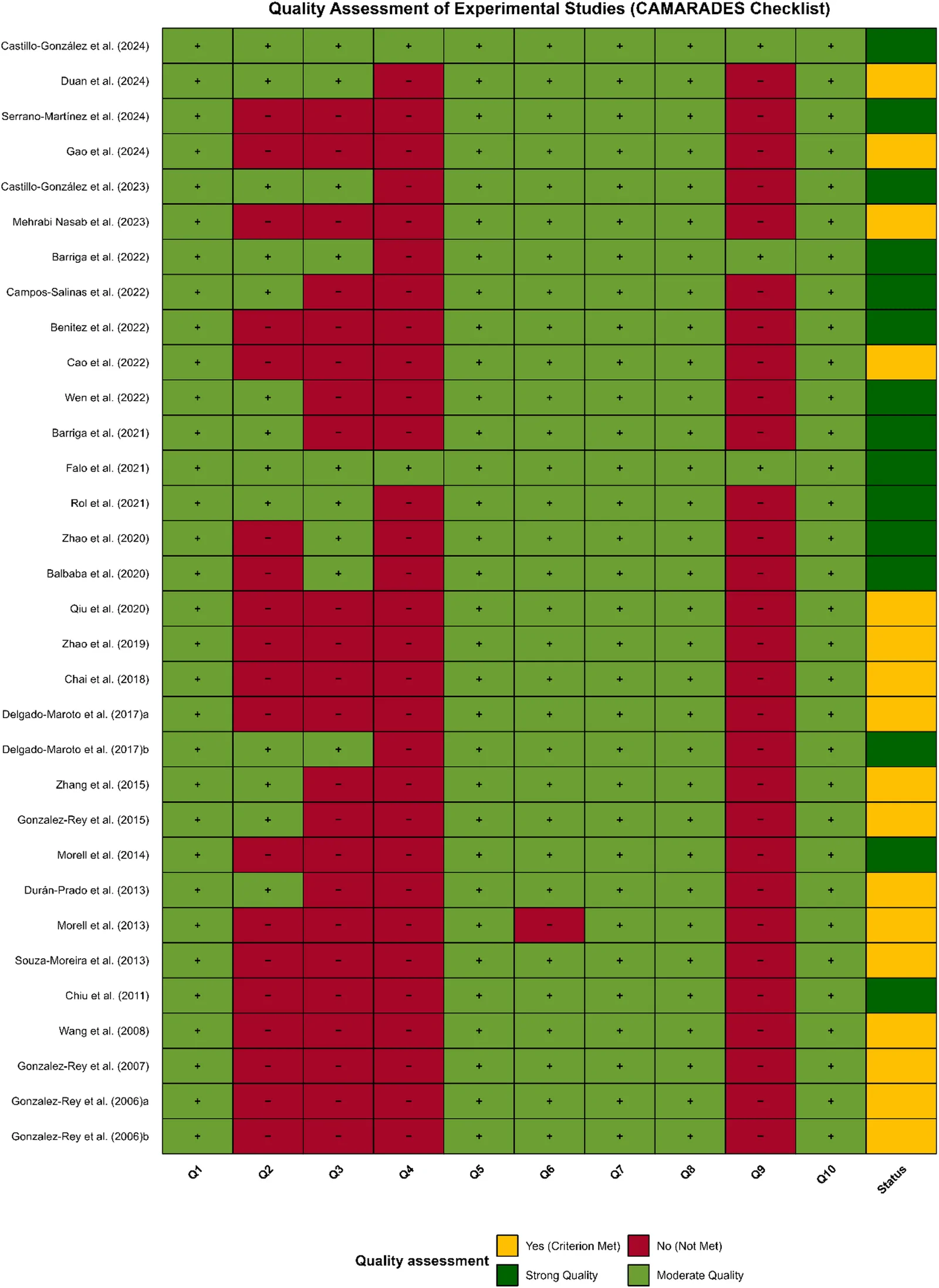
Risk of bias and methodological quality assessment of included animal studies. The risk of bias was analyzed using the CAMARADES (Collaborative Approach to Meta-Analysis and Review of Animal Experimental Studies), which evaluates ten essential areas of experimental design, including animal welfare, randomization, and conflict of interest reporting. The evaluation provides a study-by-study breakdown where green indicates a criterion was met and red marks a missing detail. While the final column shows a general trend toward moderate to strong reporting standards, the map identifies frequent gaps in formal sample size planning and blinded outcome assessment, which remain significant hurdles for transparency in the field. Q1: Publication & Reporting. Q2: Random Allocation. Q3: Blinded Assessment. Q4: Sample Size Calculation. Q5: Animal Welfare. Q6: Conflict of Interest. Q7: Results in Text. Q8: Methods Detail. Q9: Withdrawals Explained. Q10: Conclusion Reflects Purpose

Overall methodological quality was high-to-moderate. Most included studies scored between 5 and 8 on the CAMARADES quality assessment scale (Fig. 4). While reporting transparency and ethical compliance were generally adequate, specific sources of bias, such as incomplete reporting of formal sample size calculations, persist in earlier literature. Notably, research conducted within the last five years (2020–2025) demonstrates a marked improvement in methodological rigor. This trend is predominantly driven by Spanish research groups, whose recent publications frequently incorporate rigorous randomization, blinded outcome assessments, and pre-specified animal welfare protocols, thereby enhancing the internal validity of the current body of evidence (Table 2).

**Table 2:** Dynamics of cortistatin in inflammatory disease models and outcomes in mice

No	Study	Key outcomes	Main findings	Mechanism
1	Castillo-González (2024)	Infarct volume; neurological score; BBB integrity; glial activation	Time-dependent: early detrimental, late protective	Glial modulation; BBB repair; reduced immune infiltration
2	Duan (2024)	Survival; cardiac function; pyroptosis; NLRP3 activation	Improved survival and cardiac function	SSTR2–AMPK–Drp1 pathway; NLRP3 inhibition
3	Gao (2024)	Bone quality; osteoblast metabolism; apoptosis	Protection against osteonecrosis	GHSR1a–Akt survival signaling
4	Serrano-Martínez (2024)	Locomotion; dopaminergic neurons; gliosis	Reduced neuron loss and improved motor activity	Neuroinflammation suppression; BDNF induction
5	Mehrabi Nasab (2023)	Cytokines; IgE; mucus secretion; eosinophilia	Nanoparticle-CST more effective	Targeted pulmonary delivery
6	Castillo-González (2023)	BBB permeability; tight junctions; endothelial genes	CST maintains BBB homeostasis	Endothelial barrier regulation
7	Barriga (2022)	Dermal thickness; collagen; microvessels; lung fibrosis; mortality	CST deficiency exacerbates fibrosis and mortality; CST-29 treatment attenuates pathology	CTGF reduction; microvessel preservation; inhibition of fibroblast activation
8	Campos-Salinas (2022)	Survival; histopathology; fibrosis markers	Latent CST is highly effective	MMP-activated prodrug delivery
9	Benitez (2022)	Liver fibrosis; HSC activation; gene expression	CST deficiency worsens fibrosis	Inhibition of hepatic stellate cell activation
10	Cao (2022)	Osteolysis; osteoblast apoptosis; inflammation	CST protects bone tissue	TNFR1–ROS–caspase-3 signaling
11	Wen (2022)	Microglial activation; BBB disruption; cognition	Attenuated neuroinflammation and cognitive impairment	Reduced microglial activation; cytokine suppression
12	Falo (2021)	Hyperalgesia; allodynia; neurotrophic factors	Reduced neuropathic pain	Neuroregenerative and anti-inflammatory effects
13	Barriga (2021)	Lung inflammation; fibrosis; mortality	CST deficiency worsens ALI and fibrosis	Macrophage deactivation; fibroblast inhibition
14	Rol (2021)	Colitis severity; colon histology	CST analogue improved the disease	Improved pharmacokinetics
15	Qiu (2020)	Airway inflammation; CCL2; NF-κB	CST deficiency worsens asthma	CCL2/NF-κB inhibition
16	Zhao (2020)	Disc degeneration; ECM degradation	CST reduced IVDD progression	Mitochondrial ROS–NLRP3 inhibition
17	Balbaba (2020)	Uveitis score; cytokines; infiltration	Reduced ocular inflammation	TNF-α and IL-6 suppression
18	Zhao (2019)	Cartilage degradation; osteophytes	CST protects against osteoarthritis	TNF receptor signaling modulation
19	Chai (2018)	AAA incidence; macrophages; MMPs	Reduced aneurysm severity	ERK1/2 pathway inhibition
20	Delgado-Maroto (2017)	Plaque size, foam cells, T cells	Reduced atherosclerosis	Treg increase; cholesterol efflux
21	Delgado-Maroto (2017)	Cardiac inflammation; T cells	Reduced myocarditis	Th1/Th17 inhibition; Treg induction
22	Zhang (2015)	Cardiac apoptosis; ER stress markers	Protection against septic cardiomyopathy	GHSR1a-mediated ER stress inhibition
23	Morell (2014)	Nociceptive behavior; neuronal signaling	Reduced inflammatory pain	Inhibition of nociceptive signaling
24	Duran-Prado (2013)	Neointima area; SMC proliferation; stenosis; cell migration	CST deficiency exacerbates neointima; CST treatment reduces vascular stenosis	cAMP/PKA and p38-MAPK activation; Akt/ERK inhibition; Rac1 suppression
25	Souza-Moreira (2013)	EAE score; T cell responses	Treatment protective; deficiency paradoxical	HPA axis activation in deficiency
26	Morell (2013)	Hyperalgesia; nociceptor signaling	Potent analgesic effect	cAMP inhibition in nociceptors
27	Chiu (2011)	Neuronal apoptosis; cytokines	Reduced neuroinflammation	Cytokine suppression
28	Wang (2008)	Allograft survival; T cell responses	Prolonged graft survival	Th1 inhibition; Th2 tolerance shift
29	González-Rey et al. (2007a, 2007b)	Arthritis severity; cytokines	Reduced arthritis progression	Th1 suppression; IL-10 induction
30	González-Rey (2006)	Survival; cytokines; organ damage	Protection from septic shock	Inhibition of TNF-α, IL-6, and IL-12
31	González-Rey (2006)	Colitis severity: histopathology	Reduced intestinal inflammation	Th1 inhibition; Treg induction

AAA, abdominal aortic aneurysm; ALI, acute lung injury; AMPK, AMP-activated protein kinase; BBB, blood–brain barrier; CST, cortistatin; CTGF: Connective tissue growth fator; ECM, extracellular matrix; EAE, experimental autoimmune encephalomyelitis; ERK, extracellular signal-regulated kinase; GHSR1a, growth hormone secretagogue receptor 1a; HSC, hepatic stellate cell; IL, interleukin; IVDD, intervertebral disc degeneration; MMP, matrix metalloproteinase; NF-κB, nuclear factor kappa B; NLRP3, nucleotide-binding oligomerization domain-like receptor pyrin domain containing 3; ROS, reactive oxygen species; SMC: smooth muscle cells; SSTR2, somatostatin receptor 2; TNF, tumor necrosis factor; TNFR, tumor necrosis factor receptor.

## Discussion

### Mechanistic basis of cortistatin signaling

This scoping review synthesizes evidence from 31 preclinical studies examining the biological roles of cortistatin (CST) across diverse experimental disease models over nearly two decades. Despite substantial heterogeneity in disease models, including neurodegenerative, musculoskeletal, cardiovascular, and systemic inflammatory conditions, CST demonstrated a remarkably consistent protective and pleiotropic biological profile. Evidence from cortistatin-deficient (Cort^⁻/⁻^) models further reinforces its role as an endogenous regulator of immune and tissue homeostasis, as deficiency states are consistently associated with exacerbated inflammation, impaired tissue repair, and increased susceptibility to disease (Qiu et al. 2020; Barriga et al. 2021, 2022; Zhao et al. 2019; Castillo-González et al. 2023; Souza-Moreira et al. 2013; Benitez et al. 2022).

CST is encoded by the CORT gene and is processed into two principal forms: CST-14, predominantly identified in rodents, and CST-29, which is conserved across species (Delgado and Gonzalez-Rey 2017; Siehler et al. 1998). Although initially characterized due to its structural similarity to somatostatin, particularly the conserved Phe-Trp-Lys-Thr pharmacophore, CST is now recognized as a multifunctional peptide with broad systemic activity. Beyond the central nervous system, CST is expressed in monocytes, macrophages, and lymphocytes, where it acts as a paracrine and autocrine mediator dynamically regulated by cellular activation (Carniglia et al. 2017; Melmed 2009). Its expression in cardiomyocytes, vascular smooth muscle cells, and endothelial cells further supports its role in peripheral responses to injury and inflammatory stress (Duran-Prado et al. 2013).

This broad tissue distribution provides a mechanistic explanation for the consistent reduction of pathological inflammation and preservation of tissue integrity observed across experimental models. CST exerts its pleiotropic effects through engagement of multiple G protein-coupled receptor (GPCR) systems. It binds with high affinity to all five somatostatin receptor subtypes (SSTR1–5), mediating classical endocrine and anti-inflammatory effects, including inhibition of pro-inflammatory cytokine release from macrophages (Siehler et al. 1998; Robas et al. 2003). The therapeutic success of somatostatin analogues such as octreotide and lanreotide further highlights the pharmacological relevance of this receptor family (Gonzalez-Rey and Delgado 2006; Theodoropoulou and Stalla 2013).

In addition to SSTRs, CST uniquely interacts with growth hormone secretagogue receptor 1a (GHSR1a) and MrgX2 (Kreienkamp 1999; Robas et al. 2003), extending its functional repertoire beyond classical somatostatin signaling. GHSR1a signaling has been implicated in cardiovascular protection, modulation of inflammatory pathways, and cell survival. In line with the findings reported in this review, CST improved cardiac function in sepsis-associated cardiomyopathy through an SSTR2-AMPK–Drp1 signaling axis, mechanistically linked to suppression of NLRP3 inflammasome activation (Duan et al. 2024). Similarly, bone preservation in osteonecrosis models was associated with GHSR1a–Akt signaling pathways involved in cellular survival and metabolic regulation (Gao et al. 2024). MrgX2 expression in mast cells and sensory neurons connects CST to neurogenic inflammation and pain modulation, consistent with the analgesic effects observed in inflammatory and neuropathic pain models (Robas et al. 2003).

At the intracellular level, CST integrates metabolic, neural, and immune signaling pathways, providing a mechanistic framework for its pleiotropic activity (Delgado and Gonzalez-Rey 2017; Gonzalez-Rey and Delgado 2006). CST signaling involves multiple complementary molecular mechanisms. First, activation of SSTRs inhibits adenylyl cyclase, reducing intracellular cAMP and attenuating PKA-dependent transcription of pro-inflammatory genes, including those driving NF-κB activation (Olias et al. 2004; Zhang et al. 2010; Cordero et al. 2018). Second, GHSR1a-mediated activation of AMPK suppresses NLRP3 inflammasome assembly, reduces mitochondrial fission via inhibition of Drp1 phosphorylation, and limits reactive oxygen species production (Herzig and Shaw 2018; Remoortel et al. 2023; Brito et al. 2025). Third, CST acts as a partial competitive modulator of TNF receptor signaling, as demonstrated by direct interaction with TNFR1 in osteoarthritis models (Cao et al. 2022). Finally, engagement of MrgX2 contributes to neuroimmune regulation at nociceptive interfaces (Robas et al. 2003; Remoortel et al. 2023). In the central nervous system, CST reduces glial activation, preserves blood–brain barrier integrity, and limits peripheral immune cell infiltration, supporting its neuroprotective function across inflammatory and neurodegenerative models (Castillo-González et al. 2023, 2024; Wen et al. 2022; Serrano-Martínez et al. 2024; Duan et al. 2024).

### Immunomodulatory and neuroimmune integration

A central finding of this review is the consistency of CST’s immunomodulatory profile across heterogeneous disease models. CST suppresses key pro-inflammatory mediators, including TNF-α, IL-1β, IL-6, IL-12, and nitric oxide, while promoting regulatory pathways characterized by increased IL-10 production and expansion of regulatory T cells (Gonzalez-Rey et al. 2006a, 2007b; Delgado-Maroto et al. 2017a, 2017b).

These immunological effects are closely associated with metabolic reprogramming of immune cells. CST-treated models exhibit reduced oxidative stress, stabilization of mitochondrial dynamics, and activation of AMPK signaling, suggesting a shift from glycolysis-driven inflammatory phenotypes toward oxidative phosphorylation–associated regulatory states (Campos-Salinas et al. 2022; Wynn 2008). This metabolic reorientation may explain the coordinated suppression of inflammation and promotion of tissue repair observed across multiple models.

The neuroimmune dimension of CST signaling appears particularly relevant in anatomically restricted tissues. In myocarditis, for example, structural barriers limit recruitment of regulatory immune cells, impairing inflammatory resolution (Cardozo et al. 2026). CST’s capacity to enhance systemic regulatory responses and promote redistribution of Tregs to inflamed tissues may therefore represent an important mechanism facilitating immune resolution in such contexts.

### Antifibrotic activity and tissue remodeling

The antifibrotic effects of CST observed in hepatic, pulmonary, and cutaneous models align with its broader immunoregulatory function. Fibrosis typically reflects unresolved inflammation characterized by persistent TGF-β signaling and sustained fibroblast activation (Wynn 2008; Souza et al. 2025). By modulating upstream inflammatory pathways and promoting macrophage deactivation, CST shifts cytokine profiles toward resolution, thereby limiting extracellular matrix deposition and pathological tissue remodeling (Barriga et al. 2021, 2022; Benitez et al. 2022).

### Translational challenges and therapeutic strategies

Despite strong preclinical evidence, CST presents important pharmacokinetic limitations. The native peptide exhibits a short plasma half-life of approximately two minutes, restricting its therapeutic applicability in chronic conditions (Melmed 2009). To address this limitation, synthetic CST analogues incorporating D-amino acids, backbone modifications, and cyclization have been developed, demonstrating improved stability and efficacy in experimental models (Mehrabi Nasab et al. 2023).

Targeted delivery strategies further enhance translational potential. Approaches such as MMP-activated prodrugs enable site-specific release in inflamed tissues, while PEGylation, albumin fusion, and nanoparticle-based delivery systems may extend circulation time and improve tissue targeting. However, systematic comparisons of these strategies remain limited.

Another challenge relates to therapeutic timing. Early inflammatory responses are essential for host defense, whereas later phases of disease appear more responsive to immunomodulatory interventions. Defining optimal therapeutic windows will therefore be essential for successful clinical translation of CST-based therapies (Castillo-González et al. 2024).

### Conference abstracts and preliminary evidence

Although not included in the formal synthesis and risk-of-bias assessment due to the absence of peer-reviewed full publications, preliminary conference reports have provided additional support for the neuroprotective and homeostatic role of cortistatin (Falo et al. 2019; Martínez et al. 2023). These reports suggest that cortistatin deficiency may exacerbate aging-associated neuroinflammation, impair remyelination dynamics, and increase susceptibility to neurodegenerative processes in experimental models of demyelination and CNS inflammation. However, because these findings are currently limited to conference abstracts and lack detailed methodological reporting, they should be interpreted cautiously until validated in full peer-reviewed studies.

## Limitations and future directions

This review has some limitations inherent to the available evidence and the scoping design. The included studies were conducted under heterogeneous experimental conditions, including differences in animal models, disease induction protocols, cortistatin isoforms, administration routes, dosing regimens, and outcome measures, which limited direct comparability and precluded quantitative synthesis. In addition, most studies originated from a limited number of research groups, primarily in Spain and China, which may restrict the generalizability of the findings. Risk-of-bias assessment also revealed recurrent methodological limitations, including incomplete reporting of randomization, blinding, and sample size calculation, factors that may influence internal validity and potentially overestimate therapeutic effects. Finally, the exclusively preclinical nature of the evidence highlights the need for validation in more diverse experimental systems. Future studies incorporating standardized experimental designs and translational models will be essential to further clarify the therapeutic potential of cortistatin.

## Conclusion

This scoping review synthesized evidence from 31 preclinical studies investigating the role of cortistatin (CST) across diverse experimental disease models. Collectively, the findings demonstrate that CST functions as a pleiotropic endogenous regulator of immune and tissue homeostasis, consistently promoting the resolution of inflammation, preservation of tissue integrity, and modulation of both innate and adaptive immune responses. These biological effects are mediated through multiple receptor systems and signaling pathways, including somatostatin receptors, GHSR1a, and TNF receptor–related mechanisms, integrating neuroimmune, metabolic, and inflammatory signaling networks. Although the available evidence remains predominantly preclinical and heterogeneous in experimental design, the overall consistency of CST-mediated protective effects across models highlights its potential as a promising therapeutic target. Future research focusing on optimized peptide formulations, targeted delivery systems, and standardized experimental frameworks will be essential to advance CST-based strategies toward clinical translation.

## Supplementary Information

Below is the link to the electronic supplementary material.


Supplementary Material 1


## Data Availability

The data that support the findings of this systematic review are available within the article and/or its Additional Material. A complete list of included studies can be found in Tables 1 and 2.

## Declarations

### Generative AI and AI-assisted technologies

During the preparation of this work, the author(s) used ChatGPT 5.2 and Grammarly (v.1.2.158.1663) to improve the readability and language grammar of the manuscript. After using this tool/ service, the authors reviewed and edited the content as needed and took full responsibility for the content of the publication.
