# Supplementary material for: Cortistatin as a pleiotropic regulator of neuroimmune and inflammatory pathways in experimental disease: a scoping review of preclinical evidence
Source: Inflammopharmacology. 2026 Jul 1;34(8):5031–46. doi: 10.1007/s10787-026-02319-x (PMC13525017; doi:10.1007/s10787-026-02319-x)
Supplement: Supplementary file 1 [file 10787_2026_2319_MOESM1_ESM.docx]

**Cortistatin as a pleiotropic regulator of neuroimmune and inflammatory pathways in experimental disease: a scoping review of preclinical evidence**

**Running title: Cortistatin in neuroimmune regulation**

Marcelo Eduardo Cardozo¹, João Victor Cardoso dos Santos², Lilian Lacerda Bueno¹˒³, Ricardo Toshio Fujiwara¹˒³, Ana Laura Grossi de Oliveira¹, Cristiane Alves da Silva Menezes²*

¹Postgraduate Program in Infectious Diseases and Tropical Medicine, School of Medicine, Federal University of Minas Gerais, Belo Horizonte, MG, 30130-100, Brazil;
²Department of Clinical and Toxicological Analysis, Faculty of Pharmacy, Federal University of Minas Gerais, Belo Horizonte, MG, 31270-901, Brazil;
³Department of Parasitology, Institute of Biological Sciences, Federal University of Minas Gerais, Belo Horizonte, MG, 31270-901, Brazil

**Supplementary Material – S1**

**Table S1. Full Search Strategy and Database Records**

| Database | Search Strategy (Full Strings) | Filters Applied |
| --- | --- | --- |
| PubMed | ((cortistatin[tiab] OR "corti statin"[tiab] OR "CST-14"[tiab] OR CST14[tiab])) AND (inflammation[tiab] OR inflammatory[tiab] OR "infectious disease*"[tiab] OR infection*[tiab] OR fibrosis[tiab] OR fibrogenesis[tiab] OR antifibrotic[tiab] OR "connective tissue growth factor"[tiab] OR CTGF[tiab] OR CCN2[tiab] OR collagen[tiab] OR "gene expression"[tiab] OR histopatholog*[tiab]) AND (animal*[tiab] OR nonhuman[tiab] OR preclinical[tiab] OR "animal experiment*"[tiab] OR "in vivo"[tiab] OR animals[mh]) NOT humans[mh] | Publication date: 2000/01/01 to 2025/12/31; Languages: English, Portuguese, Spanish. |
| Embase | ('cortistatin':ti,ab,kw OR 'corti statin':ti,ab,kw OR 'CST-14':ti,ab,kw OR CST14:ti,ab,kw) AND ('inflammation':ti,ab,kw OR 'inflammatory':ti,ab,kw OR 'infectious disease*':ti,ab,kw OR 'infection*':ti,ab,kw OR 'fibrosis':ti,ab,kw OR 'fibrogenesis':ti,ab,kw OR 'antifibrotic':ti,ab,kw OR 'connective tissue growth factor':ti,ab,kw OR 'CTGF':ti,ab,kw OR 'CCN2':ti,ab,kw OR 'collagen':ti,ab,kw OR 'gene expression':ti,ab,kw OR 'histopatholog*':ti,ab,kw) AND ('preclinical':ti,ab,kw OR 'animal experiment*':ti,ab,kw OR 'in vivo':ti,ab,kw OR 'animal'/exp) NOT 'human'/exp | Publication years: 2000–2025; Languages: English, Portuguese, Spanish. |
| Scopus | TITLE-ABS-KEY((cortistatin OR "corti statin" OR "CST-14" OR CST14) AND (inflammation OR inflammatory OR "infectious disease*" OR infection* OR fibrosis OR fibrogenesis OR antifibrotic OR "connective tissue growth factor" OR CTGF OR CCN2 OR collagen OR "gene expression" OR histopatholog*) AND (preclinical OR "animal experiment*" OR "in vivo" OR animal* OR nonhuman)) AND PUBYEAR > 1999 AND PUBYEAR < 2026 AND (LIMIT-TO(LANGUAGE, "English") OR LIMIT-TO(LANGUAGE, "Portuguese") OR LIMIT-TO(LANGUAGE, "Spanish")) | Document type: All; Date range: 2000–2025. |
| Web of Science | TS=((cortistatin OR "corti statin" OR "CST-14" OR CST14) AND (inflammation OR inflammatory OR "infectious disease*" OR infection* OR fibrosis OR fibrogenesis OR antifibrotic OR "connective tissue growth factor" OR CTGF OR CCN2 OR collagen OR "gene expression" OR histopatholog*) AND (preclinical OR "animal experiment*" OR "in vivo" OR animal* OR nonhuman)) | Timespan: 2000–2025; Languages: English, Portuguese, Spanish; Indexes: SCI-EXPANDED, ESCI. |
| Cochrane Library | (cortistatin OR "corti statin" OR "CST-14" OR CST14):ti,ab,kw AND (inflammation OR inflammatory OR "infectious disease*" OR infection* OR fibrosis OR fibrogenesis OR antifibrotic OR "connective tissue growth factor" OR CTGF OR CCN2 OR collagen OR "gene expression" OR histopatholog*):ti,ab,kw AND (preclinical OR "animal experiment*" OR "in vivo" OR animal* OR nonhuman):ti,ab,kw | Date range: 2000–2025; Trials and Reviews. |
